# New Endoscopic Indicator of Esophageal Achalasia: “Pinstripe Pattern”

**DOI:** 10.1371/journal.pone.0101833

**Published:** 2015-02-09

**Authors:** Hitomi Minami, Hajime Isomoto, Satoshi Miuma, Yasutoshi Kobayashi, Naoyuki Yamaguchi, Shigetoshi Urabe, Kayoko Matsushima, Yuko Akazawa, Ken Ohnita, Fuminao Takeshima, Haruhiro Inoue, Kazuhiko Nakao

**Affiliations:** 1 Department of Gastroenterology and Hepatology, Nagasaki University Hospital, Nagasaki, Japan; 2 Kobayashi Internal Medicine Clinic, Kobe, Japan; 3 Digestive disease center, Showa University Northern Yokohama Hospital, Yokohama, Japan; University of Eastern Finland, FINLAND

## Abstract

**Background and Study Aims:**

Endoscopic diagnosis of esophageal achalasia lacking typical endoscopic features can be extremely difficult. The aim of this study was to identify simple and reliable early indicator of esophageal achalasia.

**Patients and Methods:**

This single-center retrospective study included 56 cases of esophageal achalasia without previous treatment. As a control, 60 non-achalasia subjects including reflux esophagitis and superficial esophageal cancer were also included in this study. Endoscopic findings were evaluated according to Descriptive Rules for Achalasia of the Esophagus as follows: (1) esophageal dilatation, (2) abnormal retention of liquid and/or food, (3) whitish change of the mucosal surface, (4) functional stenosis of the esophago-gastric junction, and (5) abnormal contraction. Additionally, the presence of the longitudinal superficial wrinkles of esophageal mucosa, “pinstripe pattern (PSP)” was evaluated endoscopically. Then, inter-observer diagnostic agreement was assessed for each finding.

**Results:**

The prevalence rates of the above-mentioned findings (1–5) were 41.1%, 41.1%, 16.1%, 94.6%, and 43.9%, respectively. PSP was observed in 60.7% of achalasia, while none of the control showed positivity for PSP. PSP was observed in 26 (62.5%) of 35 cases with shorter history < 10 years, which usually lacks typical findings such as severe esophageal dilation and tortuosity. Inter-observer agreement level was substantial for food/liquid remnant (k = 0.6861) and PSP (k = 0.6098), and was fair for abnormal contraction and white change. The accuracy, sensitivity, and specificity for achalasia were 83.8%, 64.7%, and 100%, respectively.

**Conclusion:**

“Pinstripe pattern” could be a reliable indicator for early discrimination of primary esophageal achalasia.

## Introduction

Esophageal achalasia is a rare benign esophageal motility disease (1/100000) due to impaired relaxation of the lower esophageal sphincter (LES) resulting from nerve damage [1]. The dysphagia causes food retention in the esophagus, leading to distention and severe nocturnal regurgitation. Associate complications include pulmonary complications from chronic food aspiration and esophageal carcinoma. Therefore, early detection and treatment are essential for the prevention of these complications.

Recently, novel less invasive treatment, “per-oral endoscopic myotomy; POEM”, has been introduced [2]. POEM procedure has been performed for more than 60 cases in our institution between August 2010 and October 2013. Since the introduction, 71 cases of esophageal achalasia that includes 56 patients with no previous treatment referred to our hospital. Seventeen of all 56 cases took longer than 10 years to be diagnosed as an esophageal achalasia. Some patients were not diagnosed properly even after multiple sessions of endoscopic examination. Indeed, endoscopic features including substantial dilatation with tortuosity or large amount of food remnant are listed in the characteristic indicators for achalasia. These findings are visible as its radiological manifestations and generally occur with advancement of the condition. On the other hand, achalasia patients in earlier stage do not necessarily have such typical macroscopic features. Again, discrete luminal dilatation and esophageal residue and the sensation of narrowness through esophago-gastric junction (EGJ) could be missed easily during endoscopy because of the poor reproducibility and objectivity.

Esophageal manometry provides a definitive diagnostic method for achalasia. In Japan, however, this procedure is not commonly used; instead, endoscopists take charge of the first examination via esophagogastroduodenoscopy (EGD) for symptomatic cases of achalasia. Nevertheless, there have been quite a few reports regarding endoscopic diagnostics of achalasia. Iwakiri et al reported the significance of rosette-like esophageal folds in the limited area of lower esophagus as one of endoscopic findings of achalasia [3]. In their series, EGD was subjectively performed in a conscious state requiring deep inhale, which would be almost impossible under sedation. Therefore, it is of clinical importance to find additional endoscopic appearance of esophageal achalasia to diagnose accurately and consistently especially in the patients who do not have typical macroscopic findings of the disease.

The aim of the present study was to analyze the endoscopic features of esophageal achalasia. During routine endoscopy, we frequently noticed minute stripes forming a “pinstripe pattern (PSP)” on the esophageal surface of the patients. Therefore, we compared the endoscopic findings recommended by the Descriptive Rules for Achalasia of the Esophagus published in 2012 by the Japan Society of Esophageal Diseases.

## Materials and Methods

### Patients

A total of 71 cases of esophageal achalasia underwent endoscopy at our institution between August 2010 and October 2013. This single-center retrospective study included 56 cases (male: female 24: 32) that did not receive previous treatment. The other 15 patients previously received balloon dilation, botulinum toxin injection, or surgical myotomy. Esophageal achalasia was diagnosed based on esophagogastroduodenoscopy (EGD), followed by barium esophagogram and manometry. Detailed characteristics are shown in table 1. After endoscopic examination, all patients underwent POEM procedure.

**Table 1:** Clinical features of the patients diagnosed with esophageal achalasia.

Gender	male: female	24: 32
Age	2–85 years old	(med 50, mean 53.1)
Disease history	1–55 years	(med 5, mean 10.3)
Disease type	Straight: Sigmoid	48: 8
Degree of dilation	<3.5: 3.5–6.0: >6.0 cm	17: 26: 3
LESP	26.8–180.7 mmHg	med 60.0, mean 71.7

LESP, lower esophageal sphincter pressure

med, median.

This study also included a control group (n = 60; 20 healthy subjects, 18 cases of superficial esophageal cancer, 20 cases of reflux esophagitis, and 2 cases of eosinophilic esophagitis). Written informed consent was obtained from all subjects. The study was conducted according to the provision of the Declaration of Helsinki. The study was approved by ethics committee of Nagasaki University Hospital.

### Endoscopic examination

Upper endoscopy was conducted using a high-definition white light video endoscope (GIF-H260Z, Olympus medical systems Co., Tokyo, Japan).The subjects were under conscious sedation with intravenous pethidine hydrochloride (35–70 mg, Mitsubishi Tanabe Pharmaceutical Co., Osaka, Japan) supplemented with Diazepam (5–10 mg, Takeda Pharmaceutical Co., Osaka, Japan). Peristalsis suppressive agents were not administered to allow the observation of esophageal movement including abnormal contraction. All procedures were performed by an endoscopist (HM) with sufficient experience of treating esophageal motility diseases.

Following endoscopic observation, biopsy was taken from 5 cm above the EGJ. Each specimen was fixed in 10% formalin and embedded in paraffin wax. The tissue specimens were routinely evaluated for pathological diagnosis. All specimens were evaluated by experienced gastrointestinal pathologists.

## Endoscopic Features

### Japanese guideline for esophageal achalasia

**Fig 1:**
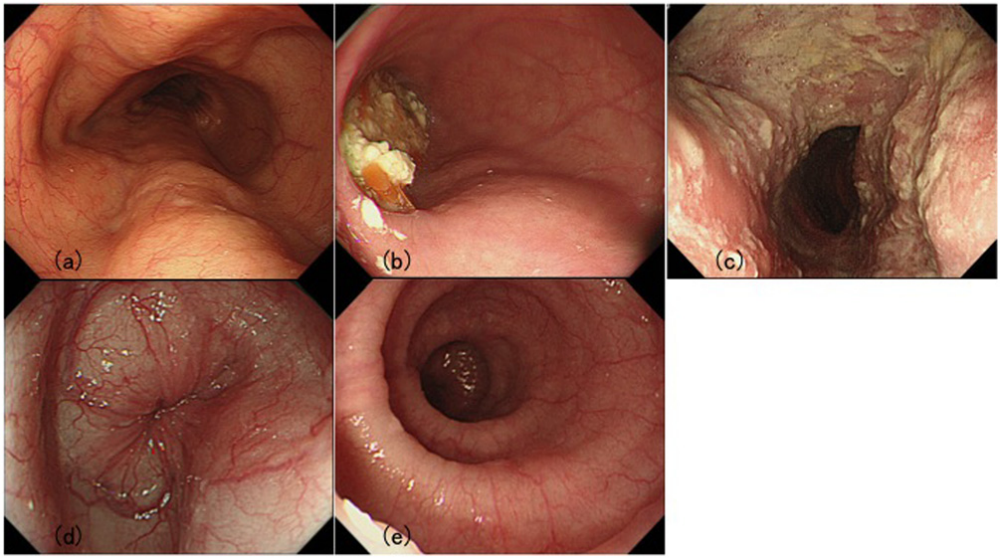
Typical findings of primary esophageal achalasia. (a) Dilation of the esophagus. Dilated esophagus drooped to both sides of the spine. (b) Food remnant in the esophagus. (c) Whitish coating of the mucosa caused by adhesion of the remained food inside of the esophagus and thickening of the mucosa. (d) Functional stenosis of the esophago-gastric junction. Endoscope passes through the tight segment with some resistance. (e) Abnormal contraction of the esophagus. Simultaneous contraction is clearly observed.

**Fig 2:**
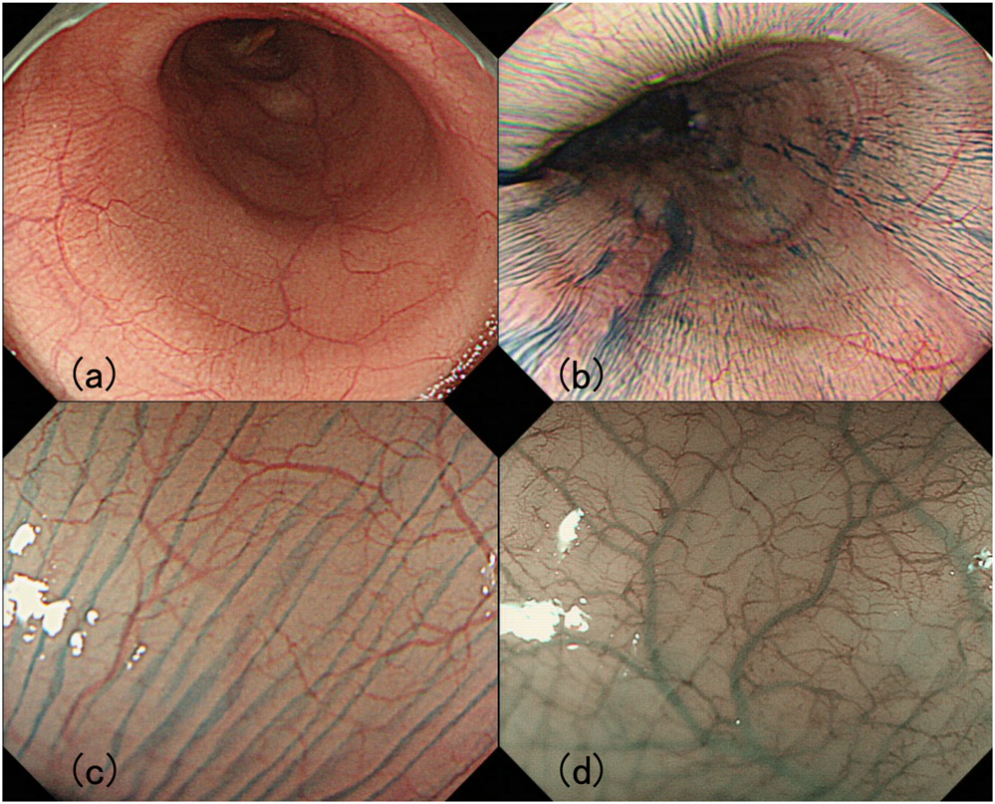
Pinstripe pattern (PSP). (a) Minute superficial wrinkle was observed on the mucosal surface. (b) Indigocarmine spraying made the superficial structure clearer. (c) Magnifying observation after indigocarmine spraying. Slight gap was observed between each longitudinal line. (d) NBI Image with magnification. The gap observed by magnifying chromoendoscopy was also identified via NBI magnification. Each superficial microvessel was substantially transparent at the gaps between stripes.

**Fig 3:**
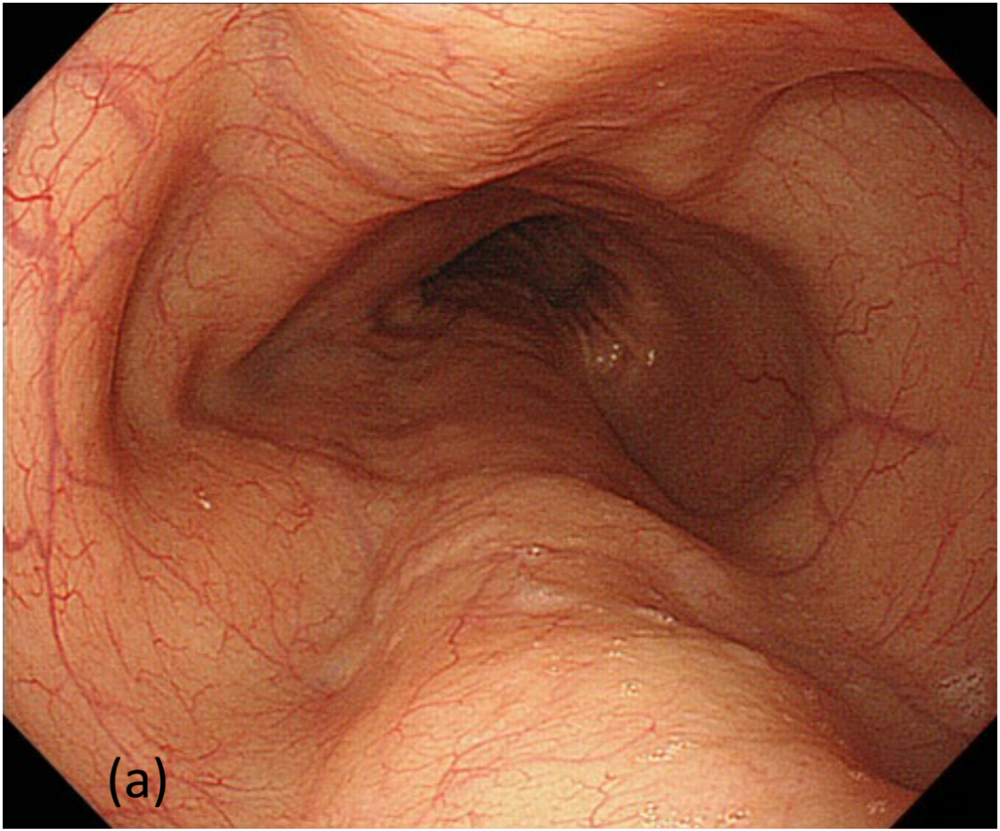
Comparison between achalasia (left) and non-achalasia (right) patients using indigocarmine and NBI.

**Fig 4:**
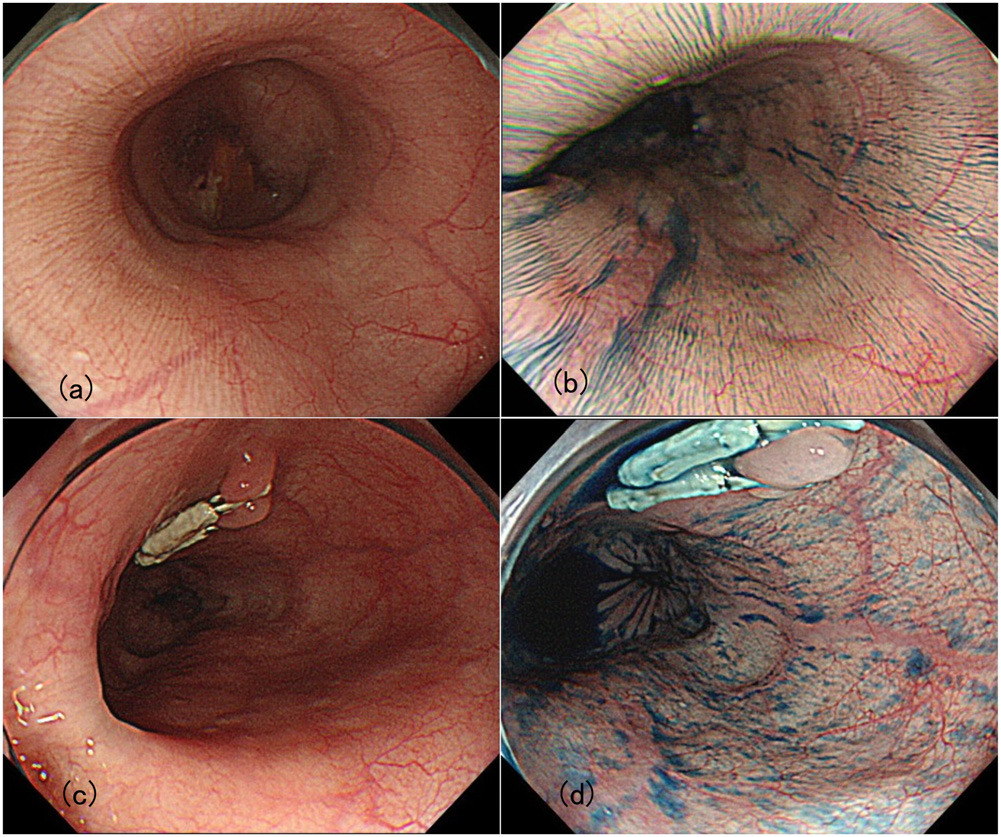
PSP change before and 3 months after POEM. (a) Conventional white light observation before POEM. Minute whitish stripe is observed on the surface of mucosa. (b) White light with indigocarmine dye. Indigocarmine emphasizes the mucosal pattern. (c) Conventional white light observation 3 months after POEM. Minute stripe was nearly disappeared. (d) Chromoendoscopy image 3 months after POEM. Minute stripe was decreased.

The Descriptive Rules for Achalasia of the Esophagus published by Japan Society of Esophageal Diseases in 2012 describe the endoscopic findings of the achalasia as follows. 1; dilatation of the esophageal lumen (Fig. 1-a), 2; abnormal liquid and/or retention of food in the esophagus (Fig. 1-b), 3; thickening and whitish change of the mucosal surface (Fig. 1-c), 4; functional stenosis of the esophago-gastric junction (Fig. 1-d), and 5; abnormal contraction of the esophageal body (Fig. 1-e).

### Pinstripe pattern, PSP

After conventional white light endoscopic observation, indigocarmine was sprayed on the mucosa surface of the entire esophagus to assess the presence of longitudinal superficial wrinkles forming a “pinstripe pattern” (Fig. 2. a–d).

### Evaluation of endoscopic features

Since this is a retrospective study, it was difficult to identify functional stenosis or abnormal contraction. Therefore, for the finding No.4, 5, we only included patients with clear description about the presence of each finding in the endoscopy reports.

Then, diagnostic agreement was met by a panel of 4 endoscopists comparing their interpretations of the endoscopic images. The total years of experience of 4 endoscopists were 3, 9, 11, and 22 years respectively.

### Statistical analysis

The inter-observer agreement levels were analyzed in 6 categories by the Fleiss’ multiple-rater Kappa analysis. The general consensus scheme for strength of agreement by κ values was used in the evaluation as follows: 0.2–0.4, fair; 0.4–0.6, moderate; 0.6–0.8, substantial; 0.8–1, excellent.

## Results

### Characteristics of the subjects

The 56 cases of esophageal achalasia included in this study (male: female, 24: 32) covered a broad range of age and disease history (Table 1). Mean duration after onset of symptoms was 10.3 years (1–55 years, median 5 years). Eight cases had sigmoid-type achalasia with a relatively long history (5–50 years, mean 20.1 years, median 20 years). The other 48 cases had straight-type achalasia with a mean disease history of 10.1 years (1–50 years, median 6 years). In most patients (36/56), the degree of dilation of the esophagus was moderate (3.5–6 cm), followed by 17 cases of mild dilation (<3.5 cm), and 3 cases of excessive dilation.

### Endoscopic features

The prevalence rate of each finding in the patients with esophageal achalasia is shown in Table 2. Functional stenosis of the EGJ was observed in 94.6% of the cases. However, dilation of esophageal lumen and food/liquid remnant in the esophagus, which are relatively clear indicators, were detected in 41.1% of the cases. Finally, PSP had a prevalence rate of 60.7% (34/56), whereas mucosal thickening and whitish change were observed in only 16.1% (9/56) of the cases. The typical PSP was only observed in patients with achalasia (Fig. 3 a, b). None of the non-achalasia patients showed positivity for PSP, including the cases of superficial esophageal carcinoma, reflux esophagitis and eosinophilic esophagitis (p < 0.001). Accordingly, the overall accuracy, sensitivity, specificity, positive predictive value, and negative predictive value of PSP for differentiating achalasia were 81.0%, 60.7%, 100%, 100%, and 73.2%, respectively. Twenty-six (62.5%) of the 35 cases that had symptoms for <10 years were positive for PSP. In addition, 20 (71.4%) of the 32 cases without food/liquid remnant in the esophagus showed PSP positivity. PSP that was observed from lower to middle thoracic esophagus disappeared or reduced in 3 to 6 months after POEM procedure in all cases (Fig. 4 a–d). Pathological examination of biopsy specimens taken from the mucosa of pre-treatment achalasia showed no significant histological change but slight thickening of the epithelium. Abnormal turnover of esophageal superficial epithelium due to persistent food stasis could be one of the reasons.

**Table 2:** Prevalence of the endoscopic findings in patients diagnosed with esophageal achalasia.

Dilation of the esophageal lumen	41.1%
Food / liquid remnant	41.1%
Mucosal thickening and whitish change	16.1%
Functional stenosis of EGJ	94.6%
Abnormal contraction of esophageal body	43.9%
Pinstripe pattern	64.7%

EGJ, esophago-gastric junction.

Multivariate statistics showed no statistical significance between each endoscopic finding and patients’ characteristics such as the disease history duration, LES pressure before the treatment, esophageal dilation grade, and subjective symptom score (Eckardt score) [4]. These indicators might be highly unreliable because of their poor subjectivity.

The inter-observer agreement level was substantial for food/liquid remnant and PSP, and fair for abnormal contraction and white coating (Table 3). The concordance rate was poor for esophageal dilatation and functional stenosis, supposedly because these parameters are affected by the skills and experience of each endoscopist.

**Table 3:** Inter-observer agreement level for each finding in patients with esophageal achalasia.

Finding	k value
Dilation of the esophageal lumen	0.0285
Food / liquid remnant	0.6861
Mucosal thickening and whitish change	0.2797
Functional stenosis of EGJ	0.0285
Abnormal contraction of esophageal body	0.2797
Pinstripe pattern	0.6098

## Discussion

The present study shows that the early diagnosis of esophageal achalasia can be very difficult without most typical findings such as extensive esophageal dilatation and massive food remnant in the esophagus. Because achalasia commonly occurs in relatively young females, these patients are sometimes misdiagnosed with psychological disorders including anorexia nervosa without proper medical assessment and examination. Furthermore, even proper examination may not detect esophageal achalasia [5]. There have been few reports focusing on endoscopic diagnosis of achalasia.

The achalasia population is also known as a high-risk group for esophageal cancer. Persistent esophageal distention causes retention of the food and liquid remnant, bacterial overgrowth, and impaired clearance of regurgitated acid and gastric contents, all leading to chronic inflammation and passively causing dysphagia and carcinoma[6] [7] [8]. In fact, 3 patients (4.2%) developed esophageal cancer in 71 achalasia patients that were admitted in our hospital. On the basis of prior data, the overall prevalence of esophageal squamous cell carcinoma in patients with achalasia has been estimated to be up to 8.6%, accounting for a 50-fold increase in cancer risk [9]. Previous studies showed that it would take on average 24 (range 10–43) years to establish carcinoma after symptom onset, and 3 to 4 years for carcinoma to develop from dysplasia [10–12]. All 3 patients who developed esophageal cancer at our institution had symptoms of esophageal achalasia for >20 years of the disease history. The recent development of less invasive and curative treatments against esophageal achalasia emphasizes the importance of early diagnosis for esophageal achalasia. Now, we insist that the significance of diagnosing achalasia in their early phases should be reconsidered.

Recent advance in the treatment of esophageal motility diseases like Heller myotomy[13] and POEM[2] captured the attentions of clinicians and the general public. When patients become aware of their dysphagia status, the first specialist they contact is generally a gastroenterologist or endoscopist.

Therefore, it is essential to establish the simple and reliable disease indicators for esophagogram and manometry. Incidentally, validating PSP requires no special skills or instruments including dye spray or magnification. PSP is detectable with white light observation, although indigocarmine can be helpful for clear recognition. This pattern is detectable even when patients are under deep sedation. Because esophageal achalasia occurs in relatively younger populations who usually require conscious sedation even for routine endoscopy examination, this offers another advantage.

The inter-observer agreement analysis revealed that the other parameters evaluated in the current report were unreliable, even food/liquid remnants (κ = 0.6861). These data are consistent with our multivariate analyses showing no statistical significance between each endoscopic finding and patients’ characteristics such as history duration, lower esophageal sphincter (LES) pressure before the treatment, esophageal dilation grade, or subjective symptom score (Eckardt score) [14]. These indicators may be highly unreliable because of their high subjectivity. However, a matched case-control study reported the presence of rosette-like esophageal folds in 97.1% of 34 patients diagnosed with achalasia without esophageal dilatation [3]. However, deep inhale is mandatory to determine the presence of rosette-like appearance, which is almost impossible under deep sedation.

The present study supports PSP for the early detection of esophageal achalasia. The pattern was observed in 73.9% (17/23) of the patients exhibiting symptoms for <5 years. In contrast, the prevalence rate was only 25.0% (2/8) for patients with a history >20 years. These patients typically exhibit severe thickening of the entire mucosal layer, including the epithelium and musclaris mucosa, with extensive submucosal fibrosis. These data suggest that PSP is related to early changes in the superficial epithelium before entire layer thickening after many years of food stasis.

Characteristic endoscopic feature of eosinophilic esophagitis is also known to be linear furrows, white plaques, and esophageal rings [15,16]. Also, wide longitudinal fold, which disappears as air is fully insufflated, can be observed. It was clearly distinguished from PSP observed in esophageal achalasia. Eosinophilic esophagitis was excluded pathologically in all the achalasia cases.

Still, the cause and the meaning of this finding are unclear. Pathological confirmation of the appearance and longer observation is awaited to elucidate the mechanism of PSP.

## Conclusion

The present study supports esophageal superficial longitudinal PSP as a possible indicator for identifying patients needing further evaluation for esophageal achalasia by esophagogram and manometry. These results are exploratory and further studies are needed in order to confirm these results.
